# Prehospital cricothyrotomies in a helicopter emergency medical service: analysis of 19,382 dispatches

**DOI:** 10.1186/s12873-019-0230-9

**Published:** 2019-01-23

**Authors:** Patrick Schober, Tessa Biesheuvel, Marcel A. de Leeuw, Stephan A. Loer, Lothar A. Schwarte

**Affiliations:** 1 Department of Anesthesiology, VU University Medical Center, De Boelelaan 1117, 1007 MB Amsterdam, The Netherlands; 2 Trauma Center, HEMS Lifeliner 1, VU University Medical Center, Amsterdam, The Netherlands

**Keywords:** Cricothyrotomy, Coniotomy, Emergency, Airway, Surgical, Prehospital, HEMS, EMS

## Abstract

**Background:**

Creating a patent airway by cricothyrotomy is the ultimate maneuver to allow oxygenation (and ventilation) of the patient. Given the rarity of airway management catastrophes necessitating cricothyrotomy, sufficiently sized *prospective* randomized trials are difficult to perform. Our Helicopter Emergency Medical Service (HEMS) documents all cases electronically, allowing a *retrospective* analysis of a larger database for all cases of prehospital cricothyrotomy.

**Methods:**

We analyzed all 19,382 dispatches of our HEMS ‘*Lifeliner 1*’, since set-up of a searchable digital database. This HEMS operates 24/7, covering ~ 4.5 million inhabitants of *The Netherlands*. The potential cases were searched and cross-checked in two independent databases.

**Results:**

We recorded *n* = 18 cases of prehospital cricothyrotomy. In all 18 cases, less invasive airway techniques, e.g., supraglottic devices, were attempted before cricothyrotomy. With exception of 2 cases, at least one attempt of orotracheal intubation had been performed before cricothyrotomy. Out of the 18 cases, 4 were performed by puncture-based technique (*Melker*), the remaining 14 cases by surgical technique. Indications for cricothyrotomy were diverse, dividable into 9 trauma cases and 9 medical cases.

The procedure was successful in all but one case (17/18, i.e., 94%; with a 95% confidence interval of 72.7–99.9%). Outcome was such that 6/18 patients arrived at the hospital alive. Long term outcome was poor, with only 2/18 patients discharged from hospital alive.

**Conclusions:**

Cricothyrotomy remains, although rare, a regularly occurring requirement in (H)EMS. Our finding of a convincingly high success rate of 94% in trained hands encourages training and a timely performance of cricothyrotomy.

## Background

Creating a patent airway by cricothryotomy or coniotomy is the last resort maneuver to allow oxygenation (and ventilation) of the patient.

In our Helicopter Emergency Medical Service (HEMS) [1], airway management is performed by physicians experienced in conventional endotracheal intubation even under suboptimal prehospital conditions [1, 2], and trained also in backup techniques, e.g., video-laryngoscopy and supraglottic devices. However, occasionally primary and backup techniques fail, rendering cricothyrotomy the last resort.

Only very few studies are available on prehospital cricothyrotomies, particularly in the civilian setting. Given the rarity of airway management catastrophes necessitating cricothyrotomy, sufficiently sized *prospective* randomized trials are difficult to perform. Our HEMS documents all cases electronically, allowing a *retrospective* analysis of a larger database for all cases of prehospital cricothyrotomies.

## Methods

We analyzed all 19,382 dispatches of our HEMS ‘*Lifeliner 1*’ since the set-up date of a searchable digital database (17.02.2011–13.05.2018). This HEMS operates 24/7, covering ~ 4.5 million inhabitants of *The Netherlands*. It is selectively dispatched to critical cases, ensuring that virtually all prehospital cricothyrotomies are performed by this HEMS.

The potential cases were searched and cross-checked in two independent databases. The first source is a nurses-serviced (Excel-)database, containing summarized case information. The second source is an extensive web-based database, filled-in by HEMS physicians, and cross-checked by a HEMS crew member. After searching the predefined *intervention* menu items in both databases, we also searched the free text entries in both databases for keyword elements ‘conio*’, ‘crico*’, ‘tracheo*’ and ‘surgical airway’ (in Dutch). In a third step, we asked all individual HEMS physicians to cross-check, whether they were aware of any cricothyrotomy cases unidentified by the digital search.

This retrospective chart review does not fall under the *Dutch Law on Medical Scientific Research Involving Human Beings* (W.M.O.), such that formal approval of the Institutional Review Board was not required.

## Results

During the 87-month study period, we registered 19,382 HEMS dispatches, of which 9130 (47%) were cancelled *en route* and 10,252 (53%) included on-site patient care. Of those cases advanced airway management was a major portion, i.e., endotracheal intubation was performed in 2502 (24%) cases.

In total, we recorded *n* = 18 cases of prehospital cricothyrotomies, corresponding to 0.18% of all non-cancelled cases. The distribution of cricothyrotomies per year was as follows: 2011: 2x; 2012: 0x; 2013: 2x; 2014: 1x; 2015: 4x; 2016: 6x; 2017: 2x; 2018: 1x. All cases were adults ≥18 years, thus there were no pediatric cricothyrotomies.

In all 18 cases, less invasive airway techniques had been performed before cricothyrotomy was attempted, e.g., facemask ventilation, supraglottic devices (laryngeal mask, i-gel®), direct laryngoscopy, or video-laryngoscopy (*Glidescope-Ranger*®). With exception of 2 cases with extremely restricted mouth opening, at least one attempt of orotracheal intubation had been performed before cricothyrotomy was attempted.

Out of the 18 cases, the first 4 (22%, years 2011–2013) were performed by a puncture-based technique (*Melker®*), where after the HEMS team switched to the surgical technique, yielding the remaining 14 cases (78%, years 2014–2018). The indications for cricothyrotomy were diverse, dividable into 9 (50%) trauma cases and 9 (50%) medical cases (Table 1).

**Table 1:** Indication for invasive airway access in *n* = 18 cases

• trauma (9 cases)	
- traffic accident: 5	
- fall from height: 2	
- other trauma causes: 2	
• non-trauma, medical (9 cases)	
- cardiovascular event: 3	
- foreign body aspiration: 2	
- anaphylaxis: 1	
- other non-trauma causes: 3	

Legend 1: The indications for the invasive, transcutaneous airway access in the *n* = 18 cases were diverse, dividable grossly into 9 trauma cases and 9 non-trauma, medical cases

The HEMS physician pool comprised on average of 12 (11–13) physicians with a low turnover. Physicians of the ‘*Lifeliner 1*’ HEMS are either staff anesthesiologists or staff surgeons. The individual HEMS physician performed on average 1.5 cricothyrotomies during the analysis period, corresponding to 0.2 cricothyrotomies per physician per year. The median number was 1 cricothyrotomy (range 0–4) per physician for this period.

The procedure was successful in all but one case (17/18, i.e., 94%; 95% confidence interval: 72.7–99.9%). Outcome was such that 6/18 patients arrived at the hospital alive. Long term outcome was poor, with only 2/18 patients (1 choking, 1 stabbing) discharged from hospital alive.

## Discussion

This study shows that cricothyrotomy, although rare, remains a regularly occurring event also in physician based (H)EMS. Introduction of video-laryngoscopy and supraglottic devices did not render this obsolete. The cricothyrotomy was successful in all but one case (94%) in establishing a patent airway, the only exception being one cardiovascular patient where the endotracheal tube repeatedly diverged cranially and tube (re-)positioning was hindered by profound hemorrhage from the cricothyrotomy incision. This overall high success rate should encourage to train and perform cricothyrotomies.

Outcome was such that 6 (33%) patients arrived at the hospital alive, however, long term outcome was poor, with 2 (11%) patients discharged from hospital alive. This discrepancy between the high success rate of the cricothyrotomies and the poor long-term outcome has several reasons: Obviously, (H)EMS arrival on scene might have been too late, or the underlying pathology too severe to improve outcome. Moreover, cricothyrotomies are regularly performed as last resort after repetitive, frustrate conventional airway maneuvers, thus possibly allowing patients to become severely hypoxic before the airway is secured. Therefore, given the high success rate of cricothyrotomies, well trained personnel might perform cricothyrotomies earlier than traditionally considered.

Various indications requiring cricothyrotomies have been published, e.g., epiglottitis, tumors, hemorrhage, angio-edema, or (neck-)trauma. We encountered prototypical indications for cricothyrotomy, and also add cases of other, unusual indications. The spectrum within our 18 cases was diverse, stressing that virtually every medical specialty may encounter patients requiring cricothyrotomy.

Multiple cricothyrotomy techniques have been published, however, none has proven optimal. These techniques can be classified in puncture-based techniques or surgical techniques. The puncture-based techniques usually require commercial kits, e.g., catheter-over-needle (e.g., QuickTrach®) or Seldinger kits (e.g., Melker). For the surgical approach also multiple variations exist, regarding incision technique (e.g., single deep incision versus two perpendicular incisions), or the use of tools to stent the scalpel incision, e.g., gum-elastic bougie, *Trousseau* dilator [3], *Frova*® catheter, or the scalpel handle [4].

Although several techniques have been compared in animals, mannequins or human cadavers [5], so far no study prospectively compared these techniques in (pre-)hospital settings. Therefore the decision which technique to use bases on suboptimal evidence. Our HEMS team switched during the study period (2014) from commercial Seldinger kits (Melker) to a surgical technique. Currently, our airway set contains a scalpel (size 15), a *Trousseau* trachea dilator [3], a *Frova*® catheter (14 F) [6] and a small endotracheal tube (6.0 mm).

Interestingly, the failure to perform a traditional oro-tracheal intubation was caused in at least three cases documented also by gastric regurgitation. Herein the medical case records explicitly mention obstruction of the motor suction devices as contributor to impossible oro-tracheal intubation. Possibly more advanced, obstruction-protected suction devices or rapidly available backup systems are required [7].

The published literature on cricothyrotomies largely comprises of studies performed in animals, manikins or cadavers [5, 8]. Patient data derive from case reports with the inherent risk of a positive publication bias, and very few case series [9, 10]. Among those case series, about half are US-army related and it remains unclear how those (combat-)series overlap, further limiting the available data pool [11–13]. In addition, cases from combat settings may markedly differ from the civilian setting [14]. The success rate of published prehospital cricothyrotomy series ranges between 67 and 100%, with all but one series reporting success rates of > 80%.

### Limitations

This study is *retrospective* in design and certain data of interest have not been systematically recorded, e.g., the times required to perform the cricothyrotomy. Therefore, we omit statistical comparisons of procedural times per technique. The small number of 18 cricothyrotomies limits statistical analysis and the resulting interpretation. Since all but one of the cricothyrotomies were successful and due to the small sample size, a formal comparison of the success rate of the techniques was not performed.

## Conclusions

A cricothyrotomy remains, although rare, a regularly occurring requirement even in physician-staffed (H)EMS. Our finding of a convincingly high success rate in trained hands encourages training and a timely performance of cricothyrotomies.

## Abbreviations

EMS: Emergency Medical Service
HEMS: Helicopter Emergency Medical Service
